# A microfocus study on basic-oxygen-furnace slag thin sections to understand the principles of vanadium incorporation

**DOI:** 10.1107/S1600577525003868

**Published:** 2025-06-06

**Authors:** Sophie Wunderlich, Sven Hampel, Dario Ferreira Sanchez, Thomas Schirmer, Ursula E. A. Fittschen

**Affiliations:** a Institute of Inorganic and Analytical Chemistry Clausthal University of Technology Arnold-Sommerfeld Str. 4 38678Clausthal Germany; b Center for X-ray and Nano Science Deutsches Elektronen-Synchrotron Notkestr. 85 22602Hamburg Germany; c Swiss Light Source Paul Scherrer Institute (PSI) 5232Villingen Switzerland; d Institute of Repository Research Clausthal University of Technology Adolph-Roemer Str. 2A 38678Clausthal Germany; University of Malaga, Spain

**Keywords:** vanadium, microXANES, microXRD, microXRF

## Abstract

Vanadium in basic-oxygen-furnace (BOF) slags is particularly interesting regarding the recovery of this element, which has been further strengthened by its inclusion in the list of critical raw materials by the European Union in 2017. As BOF slags can have a significant vanadium content, they continue to be a promising secondary vanadium source. Therefore, the binding mechanisms of vanadium in the respective slag minerals must be fundamentally understood to adapt a recovery process, which enables a sufficient yield and is environmentally friendly and resource-saving. This study presents a synchrotron chemical-imaging investigation based on a combined micro X-ray fluorescence, micro X-ray diffraction and micro X-ray absorption near-edge structure analysis, enabling new insights into vanadium incorporation in BOF slags. In contrast to the previously assumed incorporation of vanadium directly into the calcium silicates, it was shown that vanadium accumulates in their boundary regions as tetrahedral V^5+^ in a structure deviating from calcium silicates. This structure is most likely a poorly crystalline residuum consisting mainly of calcium, silicon and vanadium (and oxygen) but shows no evidence of a glass phase. Additionally, vanadium occurs as octahedral V^4+^ in calcium ferrites and as tetrahedral V^5+^ in very small quantities directly in the calcium silicates. The significant enrichment of vanadium in the boundary regions of calcium silicates together with the high oxidations states, resulting in high mobility, can be regarded as advantageous for future recycling strategies.

## Abstract 2

A synchrotron-based multi-modal approach shows the presence of a new vanadium-containing compound in basic-oxygen-furnace slags.

## 1. Introduction

In 2022 the steel industry produced 1885 million tons of crude steel worldwide, 71.5% via the integrated [blast furnace (BF) and basic oxygen furnace (BOF)] route (World Steel Association, 2023 ▸). For each ton of crude steel, approximately 100–200 kg basic-oxygen-furnace (BOF) slag is generated (Mahieux *et al.*, 2009 ▸). BOF slags are formed in a Linz-Donawitz converter under oxidizing conditions by the addition of lime and other additives and are primarily used for de­phospho­rization of the steel bath and removal of silicon. The metallurgical purpose of these slags also includes taking up ore by-elements, *e.g.* vanadium or titanium, that are still in the steel bath after the BF process, as well as protecting the steel bath from oxidation (V. D. Eisenhuettenleute, 1995 ▸). Vanadium should be particularly emphasized here, because it can be significantly enriched in BOF slags and has been listed as a critical raw material by the European Union since 2017, meaning its occurrence, extraction and availability are subject to critical parameters, like their production is limited to only a few countries, considered politically unstable and a shortage of the element in the future is assumed (European Commission, 2017 ▸). Therefore, conserving primary resources for future generations and the associated importance of recycling and using secondary raw materials should be a particular focus in relation to vanadium, and consequently in relation to vanadium-containing BOF slags.

Regarding the typically applied extraction of vanadium, from both primary and secondary resources, sodium salt roasting is applied accompanied by a certain complexity and a considerable environmental impact (Hukkanen & Walden, 1985 ▸; Li *et al.*, 2017 ▸; Gabra & Malinski, 1981 ▸). The recovery of vanadium from BOF slags, as a counterpart to naturally occurring vanadium sources, based on an efficient and environmentally friendly process can therefore be considered an overall goal. The feasibility of such a process of vanadium extraction from BOF slags depends almost exclusively on the vanadium-binding phases, the vanadium distribution in these phases and its oxidation states, strongly influencing its solubility (Wisawapipat & Kretzschmar, 2017 ▸) in which vanadium is present. Many studies have already investigated the vanadium distribution and the main carrier phases in BOF slags, mainly with respect to the leaching behaviour of vanadium and its environmental toxicity, but also with respect to recovery approaches (Aarabi-Karasgani *et al.*, 2010 ▸; De Windt *et al.*, 2011 ▸). Typical observations are that vanadium is preferentially incorporated in calcium ferrites and calcium silicates and that no clear enrichment in one specific phase is present. Regarding its oxidation state there are several options published, varying from, for example, vanadium being predominantly present as V^4+^, V^4+^ and V^5+^ to the presence of V^3+^, V^4+^ and V^5+^, simultaneously, and thus also showing the diversity of vanadium in BOF slags (Chaurand *et al.*, 2007 ▸; Preßlinger & Klepp, 2002 ▸; Hobson *et al.*, 2017 ▸).

This study aims to elucidate the vanadium distribution in BOF slags imaging the chemistry with micrometre resolution. Micro X-ray diffraction (microXRD), micro X-ray fluorescence (microXRF) and micro X-ray absorption near-edge structure (microXANES) analysis at the microXAS beamline at the Swiss Light Source are performed, to investigate the exact accumulations of vanadium in BOF slags as well as its structural influence on the respective hosting phases and its speciation. A deeper understanding of the vanadium distribution and its enrichment in BOF slags lays another foundation to ease its recovery and therefore on the one hand to conserve primary resources and to develop an alternative to energy-intensive roasting methods on the other.

## 2. Experimental

**Figure 1:**
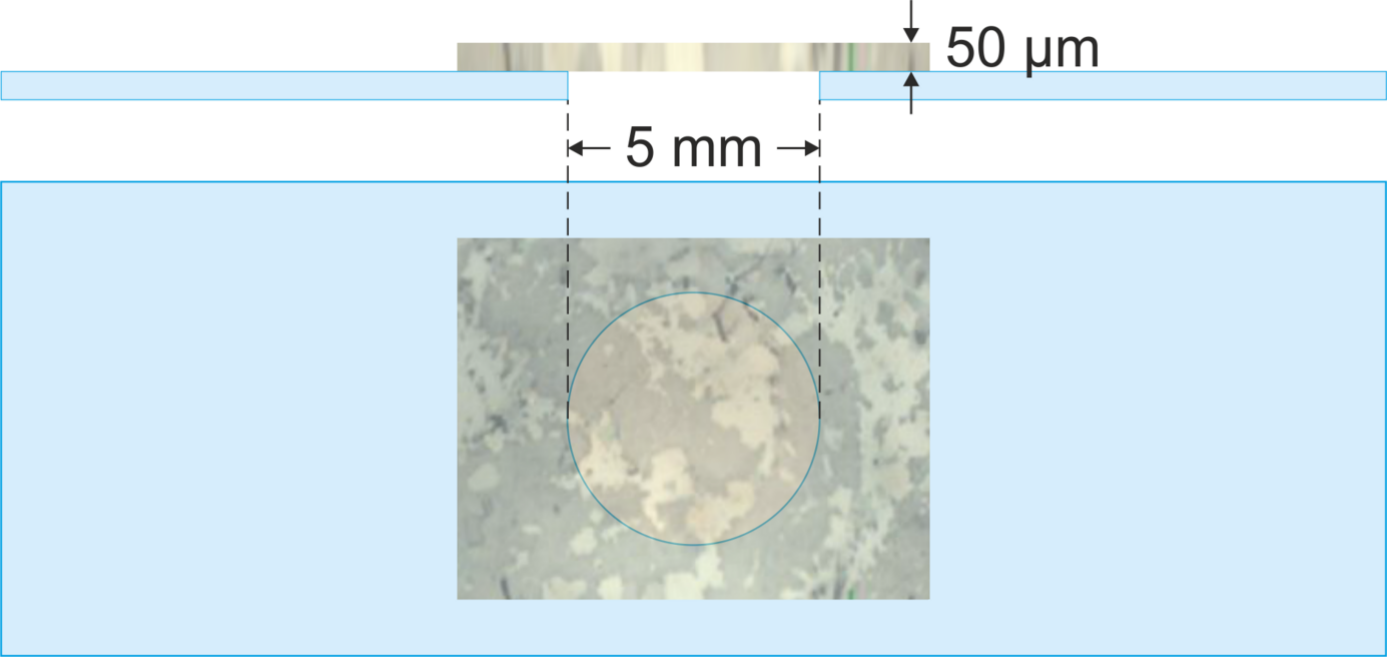
Schematic sketch of the plan view (bottom) and cross section (top) of the free-standing thin section.

The investigated high vanadium BOF slags are industrially produced slags and have been intensively investigated by electron probe microanalysis (EPMA) (Wunderlich *et al.*, 2021 ▸). For this study the samples were prepared as a thin section. The specimens (∼20 mm in diameter) were embedded in ep­oxy resin and polished down to a thickness of ∼50 µm with a final step on Pb plates. The thin section is mounted on a glass sample carrier with a hole (5 mm diameter) in the centre (Fig. 1 ▸) to allow measurements in transmission, therefore with minimized absorption or scattering effects of the glass sample carrier. Scanning regions of the free-standing film were selected by prior EPMA and laboratory microXRF (Fittschen *et al.*, 2017 ▸).

MicroXRF, microXRD and microXANES measurements were performed at the microXAS beamline at the Swiss Light Source (Paul-Scherrer-Institute, PSI, Switzerland). The spot size was set to 1 µm × 1 µm with a Kirkpatrick–Baez (KB) mirror system. A Si(111) double-crystal monochromator was used to provide an energy resolution of Δ*E*/*E* = 2 × 10^−4^. Measurements of *I*_0_ were taken with a transmission SiC diode (prototype co-developed with SenSiC) placed at the exit window of the KB mirror system, while a small SiC diode after the sample, placed in the beamstop, provided the transmission signal (as well a prototype co-developed with SenSiC).

For microXRF and microXANES in fluorescence mode, two 50 mm^2^ silicon drift detectors were placed in 45° orientation with respect to the incident beam, in backscattering position, with the sample mounted perpendicularly with respect to the incoming beam, at the X-ray focal plane. Energy calibration was performed with zirconium (for microXRF/XRD) and vanadium (for microXANES) foils. The obtained XRF spectra for microXRF and microXANES were evaluated with *pyMCA* (Solé *et al.*, 2007 ▸). The microXANES point scans were processed with a custom Python script similar to *Athena* (Ravel & Newville, 2005 ▸); 2D microXANES scans were evaluated with Python scripts, *MANTiS* (Lerotic *et al.*, 2014 ▸) for position alignment of the maps at each energy, and *TXM-Wizard* (Liu *et al.*, 2012 ▸).

A Dectris Eiger4M area detector was used for the microXRD measurements. The optics condition used allowed a high flux of about 5 × 10^10^ photons s^−1^ on the microXAS beamline, enabling fast measurements. The calibration was performed with LaB_6_. The 2D diffraction patterns were reduced to 1D by azimuthal integration with *PyFAI* (Kieffer *et al.*, 2020 ▸) and custom Python scripts for 3D cartographies output (two spatial dimensions, plus one dimension in reciprocal space, *i.e.* scattering angle 2θ) with further analysis using *ImageJ* (Schneider *et al.*, 2012 ▸). Refinement of the diffraction data was performed by Le Bail fitting or, if possible, by Rietveld refinement with the program package *Fullprof* (Rodríguez-Carvajal, 1993 ▸).

MicroXRF analysis was used to investigate the vanadium distribution in the previously selected regions and microXRD to observe the structural changes throughout these regions. MicroXRF and microXRD signals were collected simultaneously with a spot size of 1 µm × 1 µm. Multiple 2D scans were performed at 18.1 keV with a dwell time of 200 ms pixel^−1^. The sample was scanned in fly mode, which drastically minimizes the dead time.

To identify the oxidation states of vanadium present in the respective slag minerals, microXANES was performed in regions of interest selected for point scans as well as 2D scans. MicroXANES measurements for 2D scans were performed at the pre-edge area between 5.400 keV and 5.476 keV (energy steps of 0.03 keV between 5.400 keV and 5.460 keV and 0.003 keV between 5.466 keV and 5.476 keV), at the edge area between 5.481 keV and 5.491 keV (energy steps of 0.003 keV) and at the post-edge area between 5.499 keV and 5.539 keV (energy steps of 0.01 keV) with 1 s dwell time per energy point. MicroXANES measurements for point scans were performed in the range between 5.400 and 5.440 keV with energy steps of 0.005 keV, between 5.440 and 5.459 keV with energy steps of 0.001 keV and between 5.459 and 5.490 keV with energy steps of 0.0003 keV. Behind the edge the measurements were perfomed between 5.490 and 5.549 keV with energy steps of 0.001 keV, between 5.555 and 5.600 keV with energy steps of 0.005 keV and between 5.600 and 5.800 keV with energy steps of 0.02 keV. The dwell time was 1 s per energy point. Reference spectra were obtained for V_2_O_5_ (Acros, ≥98%), VO_2_ (Carl Roth, ≥99%, pure), V_2_O_3_ (Alfa Aeser, 95%), NH_4_VO_3_ (Merck, ≥99%, for analysis) and VOSO_4_·*x*H_2_O (Alfa Aeser, ≥99%, stored in a desiccator over P_2_O_5_). Each reference specimen was prepared as a pellet from 10 mg of substance and 80 mg of cellulose (microcrystalline, Merck, for spectroscopy). The substances were ground, mixed, subsequently pressed (with a MP 250 laboratory press, Maassen GmbH) to a pellet (10 t force for 2 min) and encapsulated in self-adhesive polyimide foil.

## 3. Results and discussion

From previous studies it is known that both calcium silicates and calcium ferrites play an important role with respect to the vanadium incorporation into slag minerals. While calcium ferrites typically exhibit a constant vanadium content of around 3 wt%, calcium silicates show two different modifications regarding the vanadium content: a low vanadium version with contents varying around 1 wt% and a high vanadium version with enrichments up to 18 wt% (Wunderlich *et al.*, 2021 ▸). The applied microfocus methods should both clarify the previous observations and extend them to species and structure determination. MicroXRF analysis was performed to investigate the vanadium distribution throughout the sample and was the key method for orientation on the sample. With spatially resolved microXRD analysis the structure of the vanadium-incorporating compounds was investigated, giving new insights into the internal structure of BOF slags. MicroXANES allowed the determination of the oxidation states of vanadium in the respective hosting phases.

For a better understanding of the BOF slags investigated in this study the bulk chemical composition, gained via digestion-assisted inductively coupled plasma optical emission spectroscopy analysis, is shown in Table 1 ▸.

### 3.1. MicroXRF investigations – distribution of vanadium

**Figure 2:**
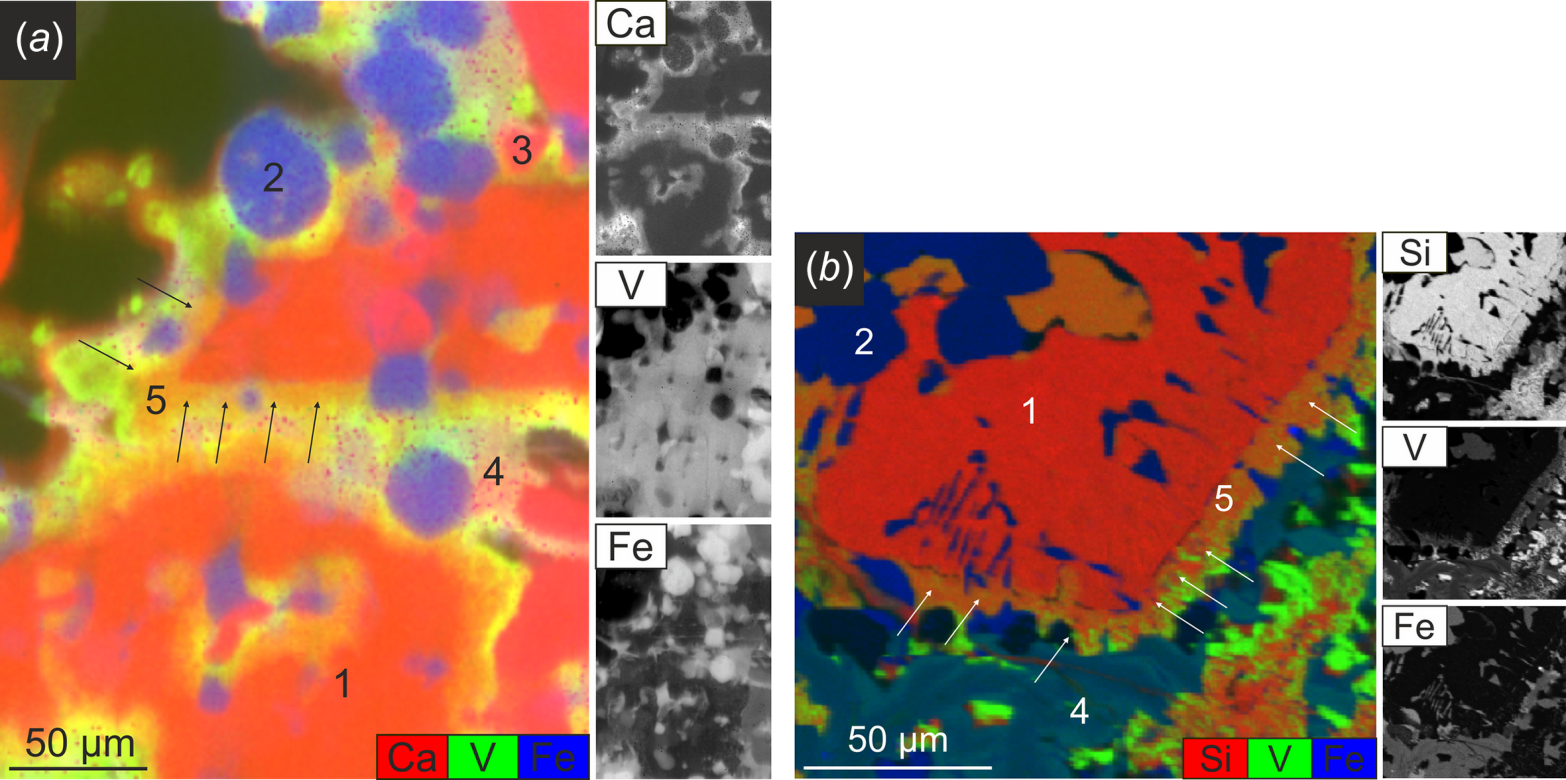
(*a*) MicroXRF data transferred to a RGB colour-coded image in a selected region and underlying element maps for calcium, vanadium and iron. (*b*) Colour-coded WDX map collected by EPMA [see World Steel Association (2023 ▸) for information regarding the method] to add silicon information to Fig. 2 ▸(*a*) and underlying element maps for silicon, vanadium and iron. #1 corresponds to calcium silicates, #2 to wüstite, #3 to calcium oxide and #4 are calcium ferrites. #5 indicates a high vanadium region [black arrows in (*a*) and white arrows in (*b*)] around grains from type #1.

From the recorded full spectra, the signal contribution of elements such as iron, vanadium and calcium were deconvolved using *PyMCA* (Solé *et al.*, 2007 ▸) – these particular three elements could be correlated to existing phases present in the samples. Fig. 2 ▸(*a*) shows the scattered intensity contributions of iron, vanadium and calcium transferred into RGB colour code, with iron indicated in blue, vanadium in green and calcium in red. Silicon, as a typical phase-forming element present in BOF slags, was not detectable during this experiment due to its low energy signal by air absorption. Therefore, Fig. 2 ▸(*b*) shows an RGB image of another thin section of the same sample containing information regarding the silicon distribution and collected by prior EPMA. In Fig. 2 ▸(*b*), iron is again shown in blue and vanadium in green; additionally silicon is shown in magenta. Grains indicated by #1 in Fig. 2 ▸ correspond to calcium silicates, #2 can be attributed to wüstite, #3 to calcium oxide, and phase #4 can be attributed to calcium ferrites. Around grains from type #1 a vanadium-rich region can be observed, labelled #5 and underlined by the arrows in Fig. 2 ▸.

### 3.2. MicroXRD investigations – structural influences of vanadium incorporation

**Figure 3:**
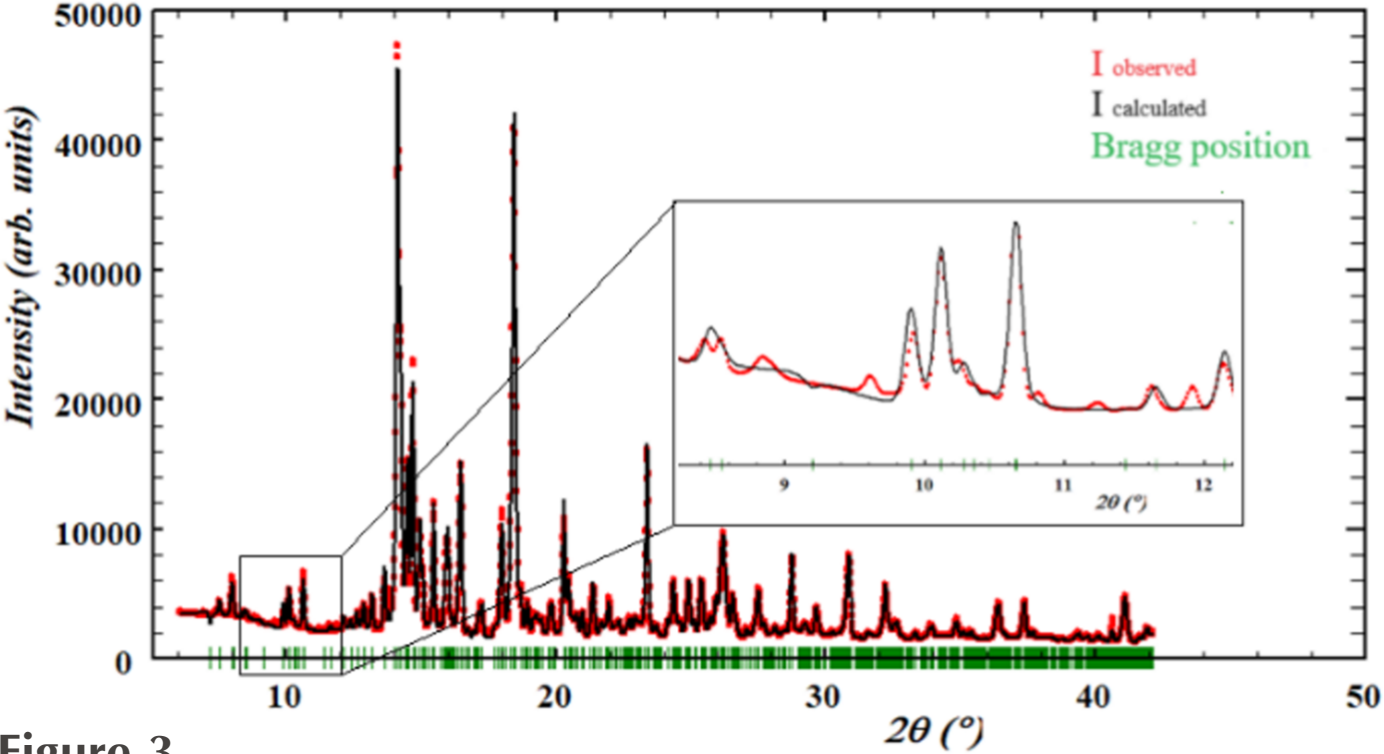
Le Bail fit of the summed diffractogram of a scanned area of 400 µm × 300 µm (λ = 0.684995 Å). The calculated intensity corresponds to the known phase content of prior PXRD measurements. The observed intensities are shown in red, the refined model in black and the respective Bragg positions in green. Due to the high resolution of microXRD, peaks that do not belong to the phase content known from prior PXRD are resolved (zoom).

MicroXRD analysis allows the crystalline structure to be examined with a spatial resolution of 1 µm. The main phase distribution of the sample is known from prior powder X-ray diffraction (PXRD), consisting mainly of 40 wt% β-Ca_2_SiO_4_, 18 wt% Ca_2_(Al,Fe)_2_O_5_, 15 wt% (Mg,Fe)O and 5 to 10 wt% CaO. Fig. 3 ▸ shows a summed microXRD diffractogram from a selected region of the sample (area 400 µm × 300 µm) fitted by the Le Bail method according to the known phase content from prior PXRD analysis. Since the sample does not fulfil ideal powder conditions, the intensity distribution cannot be regarded. The calculated intensities, shown in black (model), fit the observed intensity, shown in red (experiment), at the calculated Bragg positions. However, it can be clearly seen that there are peaks present that are not covered by the phase content known from prior PXRD (zoom). These peaks could originate from a subordinate compound that could not be resolved with PXRD and could now be made visible by the high resolution of microXRD. It is not possible to assign these peaks to a specific phase, which may be due to the fact that they are one or more only partially crystalline components.

### 3.3. MicroXANES – speciation of vanadium in respective compounds

**Figure 4:**
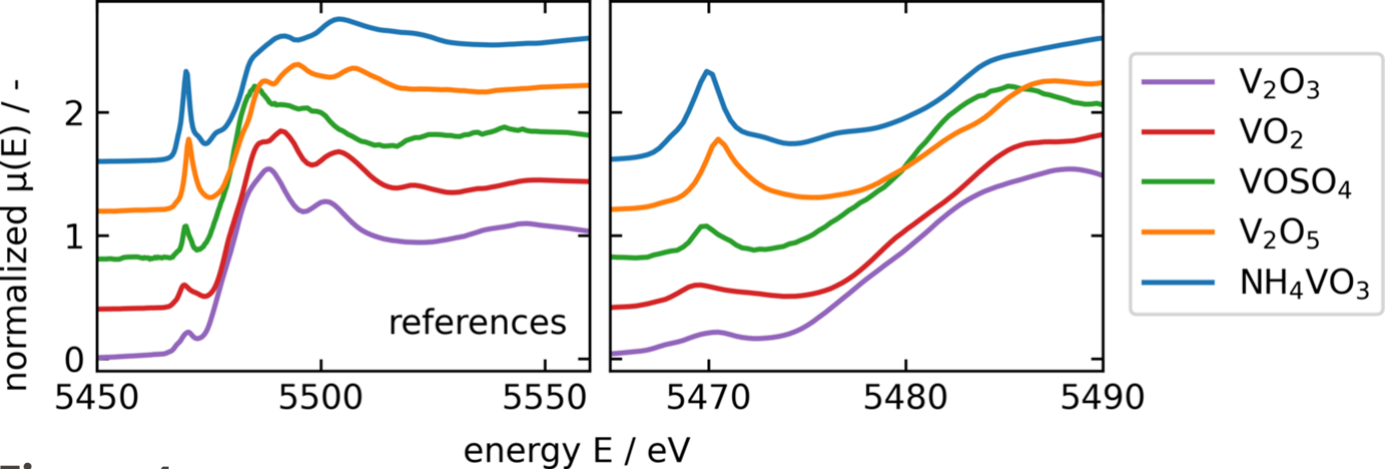
XANES spectra of the references investigated in this study. NH_4_VO_3_, tetrahedrally coordinated V^5+^, shown in blue, exhibits a pronounced pre-edge peak. This is also visible for V_2_O_5_, fivefold-coordinated V^5+^, shown in orange. V_2_O_5_ shows in comparison with NH_4_VO_3_ a slight shift of the pre-edge peak position to higher energies, which is given by the higher coordination number. Both references for sixfold coordination, V_2_O_3_, V^3+^ and VO_2_, V^4+^, are characterized by a high white-line intensity, and shift in edge position to lower energies. VOSO_4,_ shown in green, exhibits a small pre-edge peak due to its distorted octahedral coordination.

Throughout the whole sample, microXANES spectra at the V *K*-edge were collected in regions of interest (calcium silicate grains, vanadium-rich regions around calcium silicate grains, calcium ferrites), selected on the basis of prior microXRF analysis. MicroXRF showed the presence of vanadium in three different slag compounds: calcium silicates (#1 in Fig. 2 ▸), calcium ferrites (#4 in Fig. 2 ▸) and the boundary compound around calcium silicates (#5 in Fig. 2 ▸), all exhibiting different concentrations. While calcium silicates contain only minor vanadium concentrations [∼1 wt% (Wunderlich *et al.*, 2021 ▸)], calcium ferrites show slight enrichment of vanadium [∼3 wt% (Wunderlich *et al.*, 2021 ▸)]. The boundary compound around calcium silicates is significantly enriched in vanadium [∼16 wt% (Wunderlich *et al.*, 2021 ▸)]. MicroXANES at the V *K*-edge gives information regarding the respective coordination in these compounds. The measured microXANES spectra were compared with references in order to assign the observed features to defined species. References included vanadium in oxidation states V^3+^, V^4+^ and V^5+^, which are, according to the literature (*e.g.* Preßlinger & Klepp, 2002 ▸; Hobson *et al.*, 2017 ▸), most likely to occur in BOF slags and in three different coordination environments. The different references and their characteristics are summarized in Table 2 ▸ and shown in Fig. 4 ▸. Depending on the respective coordination environment, vanadium shows clear spectral characteristics before and after the *K*-edge: the pre-edge peak height and energy position, the energy position of the edge and the white line intensity. A clear pre-edge peak (caused by the symmetry-forbidden 1*s* → 3*d* transition) occurs with fourfold- or fivefold-coordinated vanadium atoms. Increasing coordination number leads to a decrease in the pre-edge peak intensity as well as to a shift of the pre-edge peak position to higher energies. Higher oxidation states in the respective coordination environment led to a shift of the *K*-edge to higher energies as well as a decrease regarding the white line intensity (Levina *et al.*, 2014 ▸).

**Figure 5:**
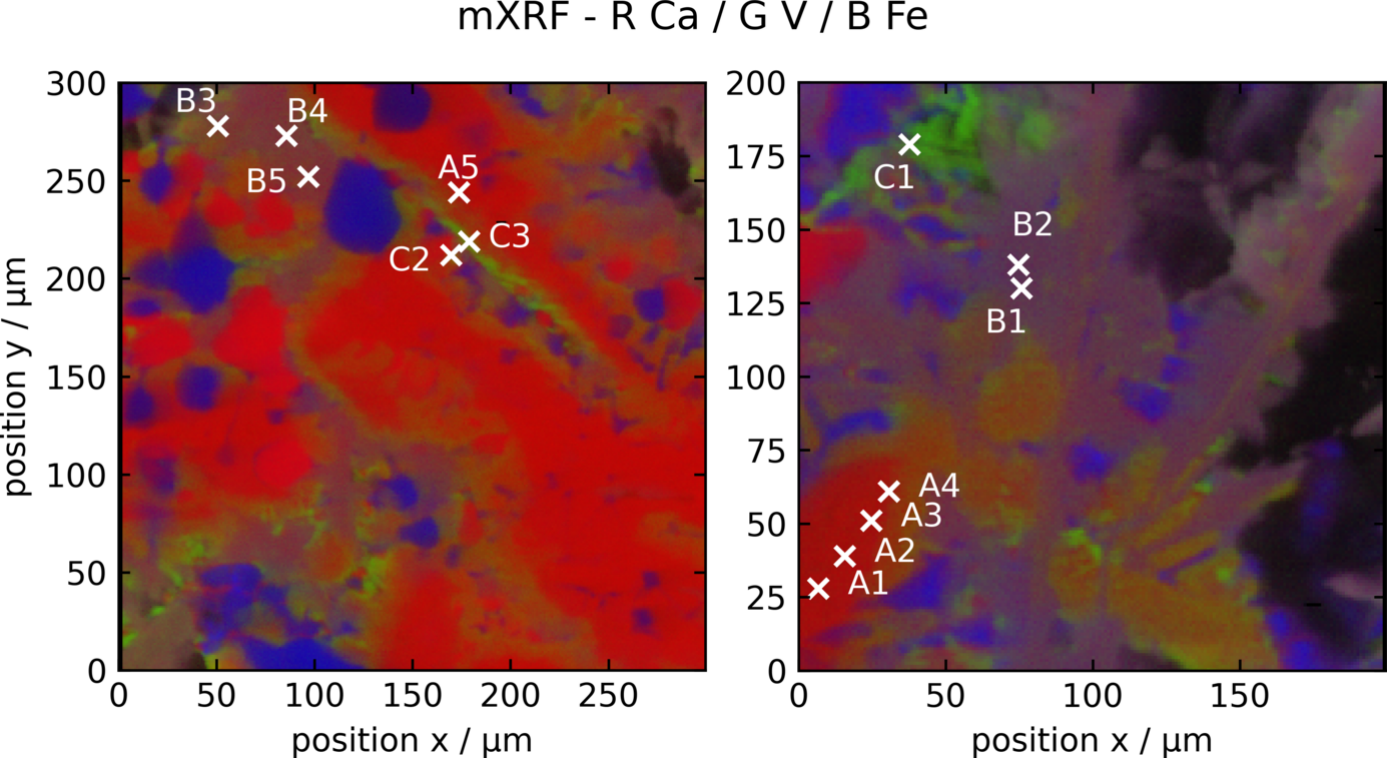
Two areas of XRF images present in the BOF slag sample with the respective measurement points for microXANES. Colour-coding: R = Ca, G = V, B = Fe.

Fig. 5 ▸ shows the microXRF maps of two areas of interest. Marked measurement points (*n* = 3) were chosen for microXANES of different vanadium species present in the BOF slag.

**Figure 6:**
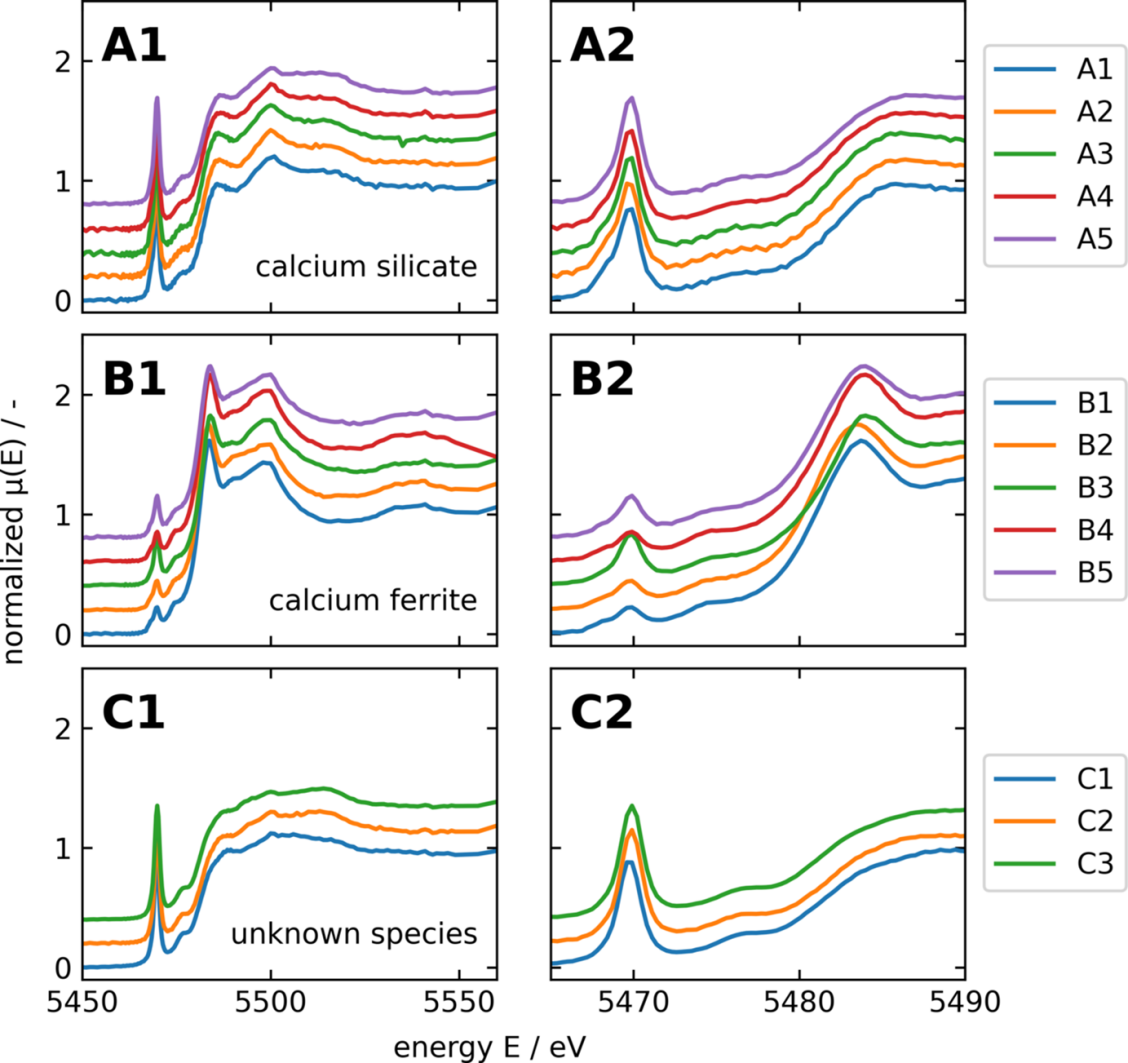
Spectra of point scans of different species (A1–A2 = calcium silicates, B1–B2 = calcium ferrites, C1–C2 = unknown species) present in the BOF slag indicated in Fig. 5 ▸.

Fig. 6 ▸ shows the XANES spectra of the microXANES of the V *K*-edge of the three slag compounds (A, B and C) incorporating vanadium. Vanadium in calcium ferrites (Fig. 6 ▸, B1–B2) can be assigned to V^4+^ and is similar to VOSO_4_, while vanadium in calcium silicates (Fig. 6 ▸, A1–A2) can be assigned to NH_4_VO_3_ based on the edge energy and the pre-edge peak and thus contains V^5+^. Species of V^3+^ could not be detected in the analysed sample. The third, unknown, species (present in boundary regions around calcium silicates, Fig. 6 ▸, C1–C2) has comparable XANES spectra with vanadium in calcium silicates. Slight deviations from the spectrum of dicalcium silicate can be observed in the area behind the edge (5485–5550 keV) and the respective features of the spectrum are less pronounced.

It is assumed that calcium silicates and calcium ferrites play a major role in the incorporation of vanadium in BOF slags (*e.g.* Preßlinger & Klepp, 2002 ▸; Spanka *et al.*, 2018 ▸; Hobson *et al.*, 2017 ▸). To estimate to what extent an impurity incorporation is possible, for a first approximation the respective ionic radii can be considered. Vanadium most likely substitutes silicon in the calcium silicates. According to Shannon & Prewitt (1970 ▸), the ionic radius for tetrahedral Si^4+^ is 26 pm, while tetrahedral V^5+^ exhibits a higher ionic radius of 35.5 pm (most likely leading to strain in the lattice). Besides vanadium, the incorporation of P^5+^ in calcium silicates is well known from the literature (*e.g.* Fix *et al.*, 1969 ▸). This co-incorporation of phospho­rous could compensate the strain induced by the larger ionic radius of V^5+^ due to a significantly smaller ionic radius of P^5+^: 17 pm, in comparison with silicon. Regarding the calcium ferrites, this study shows an incorporation of V^4+^ in octahedral coordination. Substitution of Fe^3+^ by V^4+^ in calcium ferrites is most likely due to similar ionic radii [octahedral Fe^3+^ = 55 pm, octahedral V^4+^ = 58 pm (Shannon & Prewitt, 1970 ▸)]. While some authors (*e.g.* Chaurand *et al.*, 2007 ▸; Preßlinger & Klepp, 2002 ▸) describe the presence of V^3+^ and V^5+^ or only V^5+^ or V^4+^ in BOF slags, this confirms only the presence of V^4+^ and V^5+^, which is in good agreement to the findings of, for example, Hobson *et al.* (2017 ▸). Our previous modelling approach based on EPMA data (Wunderlich *et al.*, 2021 ▸), which was applied on the samples investigated in this study, led to the hypothesis of vanadium being present in the oxidation states V^3+^ in calcium ferrites and both V^4+^ and V^5+^ in two types of calcium silicates. In contrast, the present microfocus-based investigations show higher oxidation states for all phases, compared with the model. This is realistic since in the BOF process oxygen is blown onto the melt, influencing local equilibria. Besides the major slag minerals calcium ferrites and calcium silicates as vanadium carriers, a new carrier phase was observed: in addition to the slight incorporation into calcium silicates directly (recognizable by the slight deviation from bright red in Fig. 2 ▸), a clear enrichment around these grains occurs. Throughout the entire thin section, calcium silicates exhibit a high vanadium boundary region, expanding up to 30 µm, which is of unknown origin so far. Colour-coding shown in Fig. 2 ▸ reveals the presence of calcium (red) together with vanadium (green), and according to prior EPMA analysis [Fig. 2 ▸(*b*)] silicon is also present in the vanadium enriched regions. Regarding the origin of these high-vanadium boundary regions, the following hypotheses can be established:

(1) It is a second-generation β-Ca_2_SiO_4_ (structurally similar), crystallizing at a later stage with increased vanadium content.

(2) It is a compound, structurally deviating from β-Ca_2_SiO_4_ and accumulating at their grain boundaries and preferentially incorporating vanadium, *e.g.* calcium vanadates.

(3) It is a poorly crystalline residual melt, incorporating incompatible vanadium.

To discuss the first hypothesis the solubility limits of impurity incorporation in β-Ca_2_SiO_4_ must be considered. To estimate the solubility of vanadium in β-Ca_2_SiO_4_, solubility data of phospho­rus can be used as a guide and first approximation. Phospho­rous is largely examined in BOF slags and is also incorporated in calcium silicates (Fix *et al.*, 1969 ▸; Xie *et al.*, 2012 ▸; Duée *et al.*, 2015 ▸; Suito & Inoue, 2006 ▸; Wu *et al.*, 2011 ▸). β-Ca_2_SiO_4_, which is detected by XRD in the solid state of the BOF slag, is not present at the beginning of crystallization since it is a product of solid–solid phase transformation. Looking at its origin there are two possibilities depending on the calcium-to-silicon ratio in the melt: with a calcium-to-silicon ratio of around 2:1, α-Ca_2_SiO_4_ crystallizes, which is the high-temperature polymorph of Ca_2_SiO_4_, and decomposes during cooling below 675°C (Bredig, 1950 ▸) to β-Ca_2_SiO_4_. Regarding the solubility limit of impurities in β-Ca_2_SiO_4_, originated according to this mechanism, the solubility limits in α-Ca_2_SiO_4_ need to be regarded. If the calcium-to-silicon ratio exceeds 2:1, tricalcium silicate, Ca_3_SiO_5_, crystallizes primarily, which is a high-temperature phase, and decomposes below 1250°C to CaO and Ca_2_SiO_4_ (Lea & Parker, 1934 ▸; Mohan & Glasser, 1977 ▸). Regarding the solubility limit of impurities in β-Ca_2_SiO_4_, originated according to this mechanism, the solubility limits in Ca_3_SiO_5_ need to be regarded. Since the calcium-to-silicon ratio exceeds 2:1 in the investigated BOF slags, the second option is more likely. According to Diouri *et al.* (1997 ▸) the limit of P_2_O_5_ inclusion in Ca_3_SiO_5_ is 1.1 wt% (corresponding to 0.48 wt% P). Assuming V^5+^ and P^5+^ show identical behaviour, and both substitute silicon in Ca_3_SiO_5_, the solubility limit is reached rapidly. Therefore, higher amounts of these impurities cannot be incorporated in β-Ca_2_SiO_4_ originated from Ca_3_SiO_5_ decomposition, leading to a distinctive enrichment of vanadium in the residual melt and, contradicting the hypothesis #1, the unknown high vanadium component being a second-generation β-Ca_2_SiO_4_ with higher vanadium concentration.

Regarding the hypotheses (2) and (3), *i.e.* the presence of a structurally different vanadium-incorporating compound and the presence of a poorly crystalline residual melt, the results of this study provide clear indications. Simultaneous microXRD measurements of selected scanning regions (*e.g.* area 400 µm × 300 µm) were evaluated based on prior PXRD refinements, with a known phase content composed of β-Ca_2_SiO_4_ (larnite), Ca_2_Fe_2_O_5_/Ca_2_AlFeO_5_ (srebrodolskite/brownmillerite), FeO (wüstite) and CaO (lime). The summed diffractograms collected by microXRD contain minor peaks which are not covered by the known phase content. The well known calcium vanadates, *e.g.* Ca_3_(VO_4_)_2_ monoclinic, Ca_3_(VO_4_)_2_ triclinic or CaV_3_O_7_, could be clearly excluded based on their peak positions. The significance of these peaks with respect to an unknown vanadium-incorporating structure cannot be clearly determined. Since the high resolution of microXRD analysis is not able to unambiguously specify a possible vanadium-incorporating compound by its structure, it is probable that very small grain sizes and low symmetry of the respective compound together with poor or partly crystallinity increase the difficulty in assigning a discrete compound to the observed peaks. Furthermore, an assignment is complicated by the high number of peaks belonging to β-Ca_2_SiO_4_ potentially causing overlapping. The presence of a glassy compound could be clearly excluded by the absence of amorphous halos in the diffractograms.

**Figure 7:**
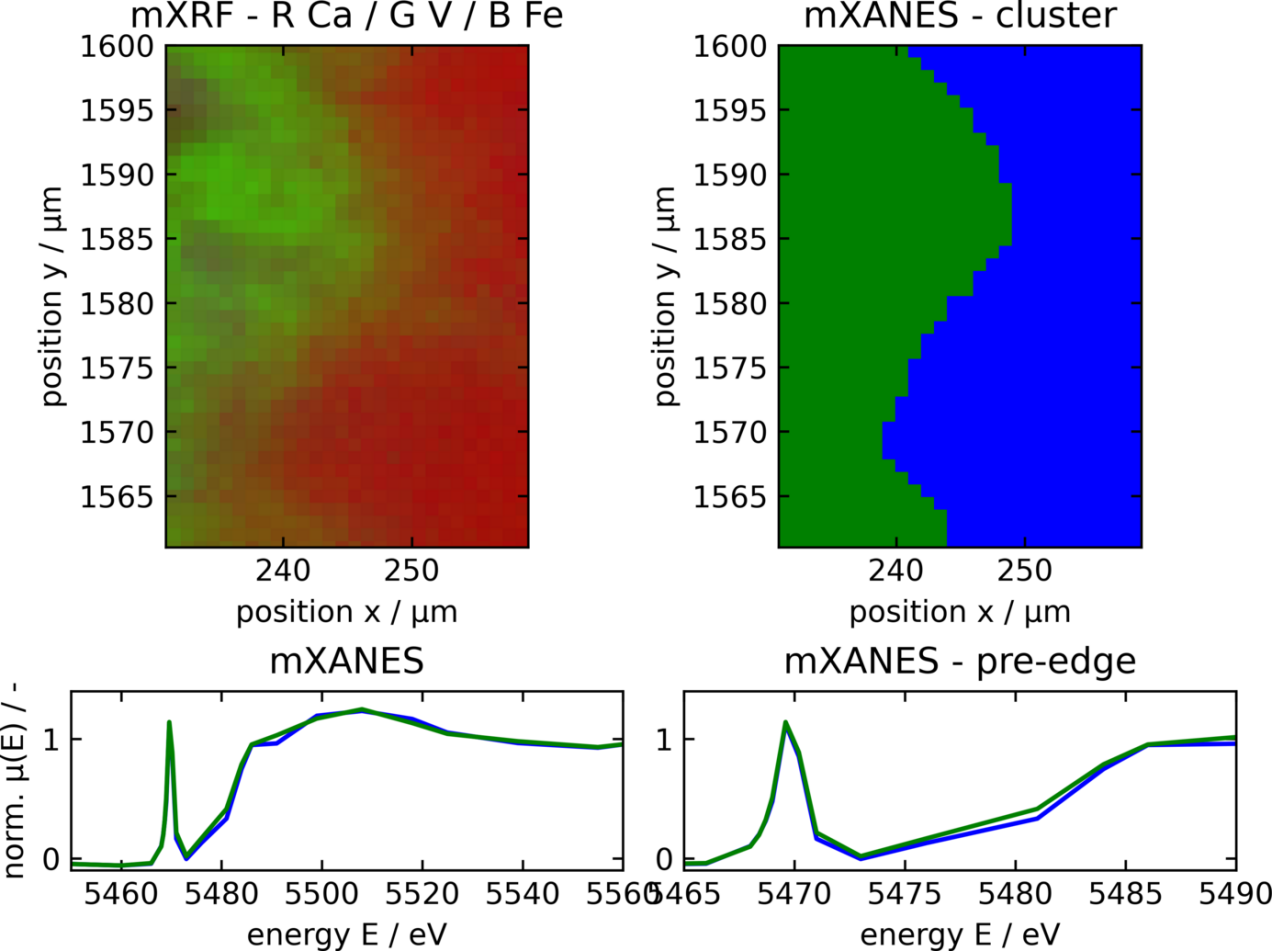
A microXRF image of a region of calcium silicates and vanadium compound around calcium silicates is presented, with colour code R = Ca and G = V (top left). The respective microXANES spectra collected in this region can be clustered according to their features, resulting in two clusters (top right). MicroXANES spectra of the two clusters are shown below, with blue corresponding to the calcium silicate and green to the vanadium compound around calcium silicates.

The microXANES spectra showed a similar speciation of vanadium in the vanadium compound around the calcium silicates as that of the calcium silicates themselves. Both matched the reference NH_4_VO_3_ and therefore most likely incorporate tetrahedral V^5+^. However, slight differences between calcium silicates and the unknown compound can be observed in the areas behind the edge, indicating slight deviations regarding the vicinity of vanadium. Fig. 7 ▸ shows an area selected by microXRF to investigate the differences in microXANES spectra of these two compounds. Clustering of the respective spectra leads to two versions: the calcium silicate spectra shown in blue and the vanadium compound spectra shown in green. Regarding the pre-edge peak, the two compounds behave exactly alike, indicating the tetrahedral coordination of vanadium. There is a slight deviation regarding the edge position. The area behind the edge of the green spectrum shows a slight dampening and broadening of the respective features when compared with the blue spectrum, which can be due to higher interatomic distances, and therefore be correlated to poorer crystallinity. The dampening of post-edge features is for example shown by El Koura *et al.* (2018 ▸) investigating dopants in anatase, indicating lower crystallinity, or by Hobson (2017 ▸) investigating vanadium in aqueous solution, indicating a less ordered environment.

Taking the solubility limit for impurities in β-Ca_2_SiO_4_ or Ca_3_SiO_5_, respectively, into account, the results from microXRD and microXANES, both indicating the poor crystallinity of the vanadium-incorporating compound, together with the spatial arrangement of the vanadium enrichments in the texture gained by microXRF, the most promising hypothesis is clearly that the vanadium-enriched compound is a poorly crystalline residual melt.

The purpose of the investigations of this study is to gain a fundamental understanding of vanadium in BOF slags in order to develop possible recovery methods. Especially regarding our finite resources and the associated efforts to increase recycling rates, slags are an important secondary raw material. According to the current state of knowledge, vanadium is incorporated as a minor element in calcium silicates as well as in calcium ferrites. The splitting into two phases in which vanadium is only present in subordinate amounts may be no optimal condition for efficient recovery. In contrast, this microfocus-based study demonstrated for the first time that vanadium is additionally significantly enriched in an individual, yet not clearly identified, compound. The total vanadium content (Table 1 ▸) is therefore divided into three phases: calcium silicates, calcium ferrites and a vanadium component, whereby a first approximation results in a ratio of about 1:1:2, according to
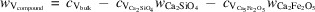
where 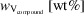 is the fraction of bulk vanadium incorporated in the vanadium compound, 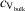 is the vanadium concentration of the bulk sample, according to Table 1 ▸ [wt%], 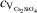 is the vanadium concentration in Ca_2_SiO_4_ [wt%],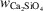 is the mass fraction Ca_2_SiO_4_ present in the sample (according to PXRD) [wt%], 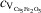 is the vanadium concentration in Ca_2_Fe_2_O_5_ [wt%] and 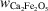 is the mass fraction of Ca_2_Fe_2_O_5_ present in the sample (according to PXRD) [wt%].

At first glance, this seems to be even worse than splitting vanadium into only two distinct components, but the significant enrichment in the vanadium compound could turn out to be a major advantage. Therefore, understanding the origin, especially the crystallization path of this vanadium compound, is particularly important in order to develop methods for proper recycling.

## 4. Conclusion

The distribution of vanadium in BOF slags is of great interest especially regarding recovery approaches of the critical raw material vanadium from steelmaking residues. In the study presented here, a combination of microXRF imaging, microXRD and microXANES methods revealed the presence of a discrete slag compound, incorporating high amounts of tetrahedrally coordinated V^5+^ and being spatially correlated to calcium silicates, for the first time. This contrasts with the previous assumption that vanadium is only incorporated directly in calcium silicates and calcium ferrites. The unknown compound seems to be a product of late crystallization accumulating incompatible ions from the residual melt and exhibiting poor crystallinity.

Although this observation of a distinct vanadium compound is a substantial gain in terms of recovery potential, questions regarding its origin and possible enrichment need to be solved. Further understanding of this compound could help develop new ideas regarding slag modifications towards a vanadium-incorporating primary crystallizing component and therefore generate a major advantage in recovery processes. Further investigations on synthetic samples, in which the amount of the vanadium-rich compound is deliberately increased, are necessary. Nevertheless, microXRF imaging combined with microXRD and microXANES has proven to be a powerful tool to locate and investigate minor compounds in multiphase systems and was able to show the behaviour of vanadium in BOF slags from a completely new point of view.

## Figures and Tables

**Table 1:** Bulk chemical analysis [mean, standard deviation (SD) and confidence interval (CI) (95%)] of the investigated BOF slag (wt%); *N* = 4 [from Wunderlich *et al.* (2021 ▸)]

	Mean	SD	CI
Al	0.60	0.06	0.09
Ca	29.7	0.3	0.4
Cr	0.48	0.05	0.08
Fe	15.8	0.3	0.5
Mg	5.18	0.16	0.25
Mn	2.23	0.02	0.03
P	0.221	0.012	0.019
Si	5.26	0.15	0.23
Ti	0.76	0.02	0.03
V	1.90	0.04	0.06

**Table 2:** Five references used in this study for comparison with respective measurements and their structural characteristics

	V oxidation state	V coordination
V_2_O_3_	V^3+^	Octahedral (Scordari, 1992 ▸)
VO_2_	V^4+^	Octahedral (Scordari, 1992 ▸)
VOSO_4_	V^4+^	Distorted octahedral (Fehrmann *et al.*, 1989 ▸; Tachez *et al.*, 1979 ▸)
V_2_O_5_	V^5+^	Fivefold (Nabavi *et al.*, 1990 ▸)
NH_4_VO_3_	V^5+^	Tetrahedral (Nabavi *et al.*, 1990 ▸; Pérez-Benítez & Bernès, 2018 ▸)
